# Matrigel Tunes H9 Stem Cell-Derived Human Cerebral Organoid Development

**DOI:** 10.3390/organoids2040013

**Published:** 2023-10-05

**Authors:** R. Chris Estridge, Jennifer E. O’Neill, Albert J. Keung

**Affiliations:** 1 Department of Chemical & Biomolecular Engineering, North Carolina State University, Raleigh, NC 27606, USA; 2 Genetics Program, Department of Biological Sciences, North Carolina State University, Raleigh, NC 27606, USA; jeoneill@alumni.ncsu.edu

**Keywords:** cerebral organoids, Matrigel, extracellular matrix, development, neurodevelopment, bioengineering, human, choroid plexus, differentiation

## Abstract

Human cerebral organoids are readily generated from human embryonic stem cells and human induced pluripotent stem cells and are useful in studying human neurodevelopment. Recent work with human cerebral organoids have explored the creation of different brain regions and the impacts of soluble and mechanical cues. Matrigel is a gelatinous, heterogenous mixture of extracellular matrix proteins, morphogens, and growth factors secreted by Engelbreth-Holm-Swarm mouse sarcoma cells. It is a core component of almost all cerebral organoid protocols, generally supporting neuroepithelial budding and tissue polarization; yet, its roles and effects beyond its general requirement in organoid protocols are not well understood, and its mode of delivery is variable, including the embedding of organoids within it or its delivery in soluble form. Given its widespread usage, we asked how H9 stem cell-derived hCO development and composition are affected by Matrigel dosage and delivery method. We found Matrigel exposure influences organoid size, morphology, and cell type composition. We also showed that greater amounts of Matrigel promote an increase in the number of choroid plexus (ChP) cells, and this increase is regulated by the BMP4 pathway. These results illuminate the effects of Matrigel on human cerebral organoid development and the importance of delivery mode and amount on organoid phenotype and composition.

## 1. Introduction

Human cerebral organoids are readily generated from human embryonic stem cells and human induced pluripotent stem cells and are useful in studying human neurodevelopment. As with any experimental model, there are several goals: to improve their reproducibility, complexity, and fidelity to in vivo tissues; to understand how components and protocol parameters control model phenotypes; and to expand the range of phenotypes that can be captured by the model. Recent work with human cerebral organoids has explored the creation of different brain regions [1–7] and the impacts of soluble [8,9] and mechanical cues [10,11].

Matrigel is a gelatinous, heterogenous mixture of extracellular matrix proteins, morphogens, and growth factors secreted by Engelbreth-Holm-Swarm mouse sarcoma cells [12]. Previous work has examined and quantified Matrigel components [13,14], but due to the complexity of Matrigel, it has been difficult to create a synthetic replacement. It is a core component of almost all cerebral organoid protocols, generally supporting neuroepithelial budding and tissue polarization [11,15,16]; yet, its roles and effects beyond its general requirement in organoid protocols are not well understood [14,17,18], and its mode of delivery is variable, including the embedding of organoids within it or its delivery in soluble form [1,11,19–22]. To our knowledge, the effects of Matrigel dosage and mode of delivery on human cerebral organoids (hCOs) have not been reported.

Given its widespread usage, we asked how H9-derived hCO development and composition are affected by Matrigel dosage and delivery method. We found Matrigel exposure influences organoid size, morphology, and cell type composition. We also showed that greater amounts of Matrigel promote an increase in choroid plexus (ChP) cells, and this increase is regulated by the BMP4 pathway. These results illuminate the effects of Matrigel on H9-derived human cerebral organoid development and the importance of delivery mode and amount on organoid phenotype and composition.

## 2. Materials and Methods

### 2.1. hESC Cell Lines

H9 (WA09; WiCell, Madison, WI, USA) were grown in E8 media (STEMCELL Technologies, Vancouver, BC, Canada) in 6-well culture dishes (Greiner Bio-One, Kremsmünster, Austria) coated with 0.5 μg/mL Vitronectin (STEMCELL Technologies). Cells were passaged every 3–5 days as necessary using 0.5 mM EDTA (Invitrogen, Waltham, MA, USA).

### 2.2. Solubilized Matrigel

Matrigel (Corning, Corning, NY, USA) was allowed to thaw on ice for 1–2 h until liquid, while 50 mL falcon tube aliquots of cerebral organoid differentiation media were allowed to warm to room temperature. Matrigel was then added directly to the room-temperature media and pipetted up and down thoroughly (~20 times) to mix.

### 2.3. Cerebral Organoid Generation

hCOs were generated through modifying the Lancaster whole brain organoid protocol [1,2]. Stem cells were grown to 75% confluency before dissociation into a single cell suspension using EDTA and Accutase (Invitrogen). 9000 cells were plated into a low-attachment U-bottomed 96-well plate (Corning) in hESC media supplemented with 50 μM Y-27632 (LC Labs, Woburn, MA, USA) and 4 ng/mL β-FGF (Gibco, Waltham, MA, USA). hESC media contained DMEM-F12 (Gibco), 20% *v*/*v* knockout serum replacement (Gibco), 3% *v*/*v* fetal bovine serum (Corning), 1% *v*/*v* MEM-NEAA (Gibco), 1% *v*/*v* Glutamax (Gibco), and 7 μL/L β-mercaptoethanol (Amresco, Solon, OH, USA). After 48 h, half of the media was replaced with hESC media containing Y-27632 and β-FGF. After another 48 h, half the media was removed again and replaced with hESC media without Y-27632 and β-FGF. On day 6, each organoid was transferred to its own well of a 24-well ultra-low-attachment plate (Corning) with 500 μL of neural induction media containing DMEM-F12, 1% *v*/*v* N2 supplement (Gibco), 1% *v*/*v* MEM-NEAA, and 1% *v*/*v* Glutamax, and 50 μg/mL heparin (Sigma, St. Louis, MO, USA). On days 8 and 10, an additional 500 μL of media was added to each well. At day 11, media were removed and replaced with cerebral organoid differentiation media without vitamin A consisting of 50:50 DMEM-F12: Neurobasal (Gibco) mixture, 0.5% *v*/*v* N2 supplement, 1% *v*/*v* Glutamax, 0.5% *v*/*v* MEM-NEAA, 1% B27 supplement w/o vitamin A (Gibco), 1% *v*/*v* Penicillin/Streptomycin (Lonza, Walkersville, MD, USA), 3.5 μL/L β-mercaptoethanol, 12.5 μL/L 4 mg/mL Insulin (Gibco), and solubilized Matrigel (Corning). The plates were immediately placed on an orbital shaker at 105 RPM to prevent organoids from settling into the Matrigel on the plate surface. Media were replaced with fresh cerebral organoid differentiation media without vitamin A after 48 h. After an additional 48 h, media were replaced with cerebral organoid differentiation media with vitamin A, which contained same components except the B27 supplement w/o vitamin A is replaced with B27 supplement with vitamin A (Gibco). Media were replaced with fresh cerebral organoid differentiation media with vitamin A every 2–3 days as necessary. Matrigel was added to fresh media until the targeted number of applications for a condition had been achieved.

### 2.4. Cryosectioning and Immunohistochemistry

Tissues were fixed in 4% paraformaldehyde (Sigma) for 15 min at 4 °C followed by three 10 min PBS washes (Gibco). Tissues were placed in 30% sucrose overnight at 4 °C and then embedded in 10% gelatin/7.5% sucrose (Sigma) in PBS. Embedded tissues were flash-frozen in an isopentane (Sigma) bath between −50 and −30 °C and stored at −80 °C. Frozen blocks were cryosectioned (Epredia, Kalamazoo, MI, USA) to 30 μm. For immunohistochemistry, sections were blocked and permeabilized in 0.3% Triton X-100 (Sigma) and 5% normal donkey serum (VWR) in PBS. Sections were incubated overnight with primary antibodies: rabbit anti-TUJ1 (1:100, Sigma T2200), goat anti-SOX2 (1:20, R&D Systems AF2018), mouse anti-Phospho-Vimentin (1:200, MBL International D076-3S), rabbit anti-EMX1 (1:50, Atlas Antibodies HPA006421), mouse anti-SATB2 (1:100, Abcam ab51502), mouse anti-Calretinin (1:100, Sigma MAB1568), rabbit anti-FOXG1 (1:1000, Abcam ab18259), sheep anti-TTR (1:100, Bio-Rad, AHP1837), mouse anti-TTR (1:100, R&D Systems, MAB7505), and rabbit anti-AQP1 (1:200, Abcam ab15080) in 0.3% Triton X-100, 5% normal donkey serum in PBS at 4 °C. Sections were then incubated with Alexa Fluor 488 and 647 conjugated secondary antibodies (1:250) in 0.3% Triton X-100 and 5% normal donkey serum in PBS for 2 h at RT, and nuclei were stained with 300 nM DAPI (Invitrogen). Slides were mounted using ProLong Antifade Diamond (Thermo Fisher, Waltham, MA, USA).

Images were taken using a Nikon AR confocal laser scanning microscope (Nikon, Tokyo, Japan). All samples within quantification experiments were imaged using the same laser intensity settings using the 10X, 20X, and 40X objectives. Quantifications were performed manually in FIJI. Organoid condition names were first removed, and the images randomized before quantification to avoid bias. A 10,000 by 10,000 grid was overlaid over the image and each grid was manually counted as positive for containing tissue if the grid contained DAPI+ cells. These grids were then remeasured, and regions were considered positive if TTR+ staining was present in the grid. At least 4 organoids per condition were collected and measured.

For cell density measurements, a region of interest was generated around the organoid DAPI outline. The area was then calculated. Cell density was measured through the calculation of the area fraction of DAPI+ to the total organoid area. At least 3 organoids per condition were collected and measured. For rosette measurements, a region of interest was generated around the organoid SOX2+ rosette outline. The area was then calculated. The average size of all rosettes from an organoid was calculated. At least 2 organoids per condition were collected and measured. For cell death measurements, a region of interest was generated around the organoid DAPI outline. The area of both DAPI and Caspase-3 within the organoid were then calculated. Finally, the cell death was calculated from the area fraction of Caspase/DAPI. At least 3 organoids per condition were collected and measured.

### 2.5. Granular Surface Phenotype Quantification

Images for size experiments were taken using an epifluorescence microscope with a documentation camera (Nikon). Quantifications were performed manually in FIJI. Organoids exhibiting more than 5 changes in curvature (2nd derivative) per 1 mm of the organoid edge were considered granular.

### 2.6. Inhibitor Treatment Experiments

hCOs were treated with either a physiologically relevant 2 μM dose of FAK Inhibitor 14 [23,24] (Tocris Biosciences, Bristol, UK), 100 ng/mL DKK-1 [25,26] (Peprotech, Cranbury, NJ, USA), 200 ng/mL of Noggin [27,28] (Peprotech), or an ultrapure water control (Invitrogen). Organoids were continually exposed to compounds as long as Matrigel remained in the media.

Images for size experiments were taken using an iPhone 11 Pro (Apple, Cupertino, CA, USA). All samples within quantification experiments were imaged using the same lighting and 1X zoom. Quantifications were performed manually in FIJI.

### 2.7. Statistical Analysis

All statistical comparisons were performed using one-way ANOVA with Tukey–Kramer post hoc analysis. The results were considered statistically significant if *p*-values < 0.05.

## 3. Results

**Figure 1.**
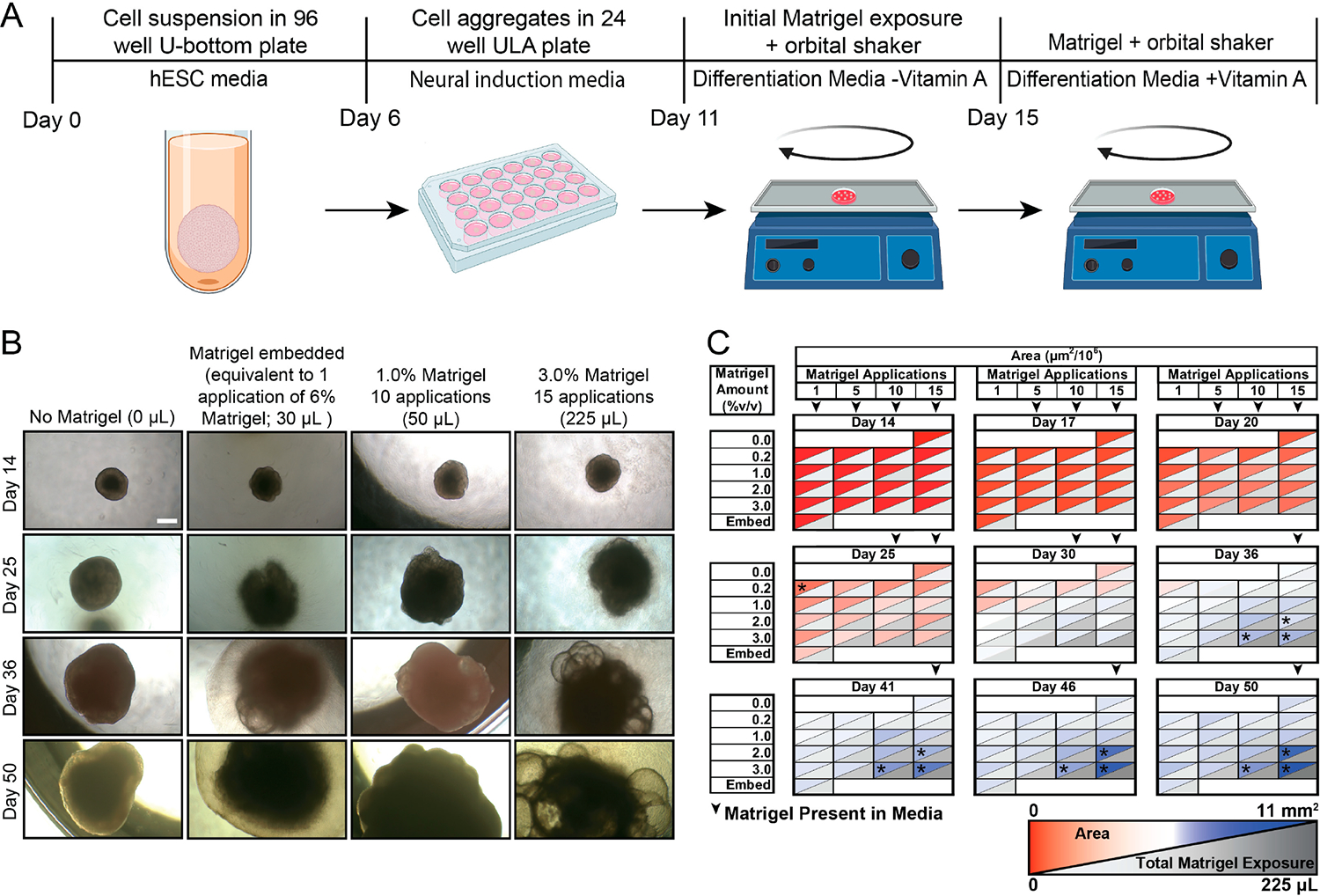
Amount of Matrigel in solution increases organoid size. (**A**) Schematic of experimental protocol, adapted from Lancaster and colleagues [15,22]. (**B**) Brightfield images of organoids with multiple Matrigel delivery regimens over a 50-day time course. Total Matrigel exposure shown in parentheses. Scale bar = 500 μm. (**C**) Summary of hCO size measurements through time course experiment (mm^2^). * *p* < 0.05, via one-way ANOVA relative to embedded (30 μL) with Tukey–Kramer post hoc analysis, n ≥ 8 organoids per condition.

Lancaster and colleagues developed a protocol for generating “whole brain” hCOs [15,22]. In this protocol, early organoids (<11 days) are embedded within Matrigel. In contrast, Velasco and colleagues added Matrigel in solubilized form when generating cortical hCOs. As Matrigel embedding is one of the most labor- and time-intensive steps of the whole brain hCO protocol, we asked if Matrigel could be added directly to the media in solubilized form and what concentration ranges would be appropriate. We generated hCOs following the Lancaster protocol [15,22] until Matrigel embedding (Figure 1A). Instead of embedding the hCOs in 30 μL of Matrigel per the protocol, we added Matrigel directly to the cerebral organoid differentiation media at concentrations ranging from 0 to 3% *v*/*v* and lengths of time (in number of applications or media changes) ranging from 2 to 39 days beginning on day 11. Media were changed every 2–3 days. Due to differences in both concentrations and lengths of time, the total volumes of Matrigel added are also reported. Organoid size was significantly affected by the Matrigel amount added (Figure 1B,C). hCOs exposed to higher amounts of Matrigel in solution were larger compared to those embedded in Matrigel and to those not exposed to any Matrigel (Figure 1B,C).

**Figure 2.**
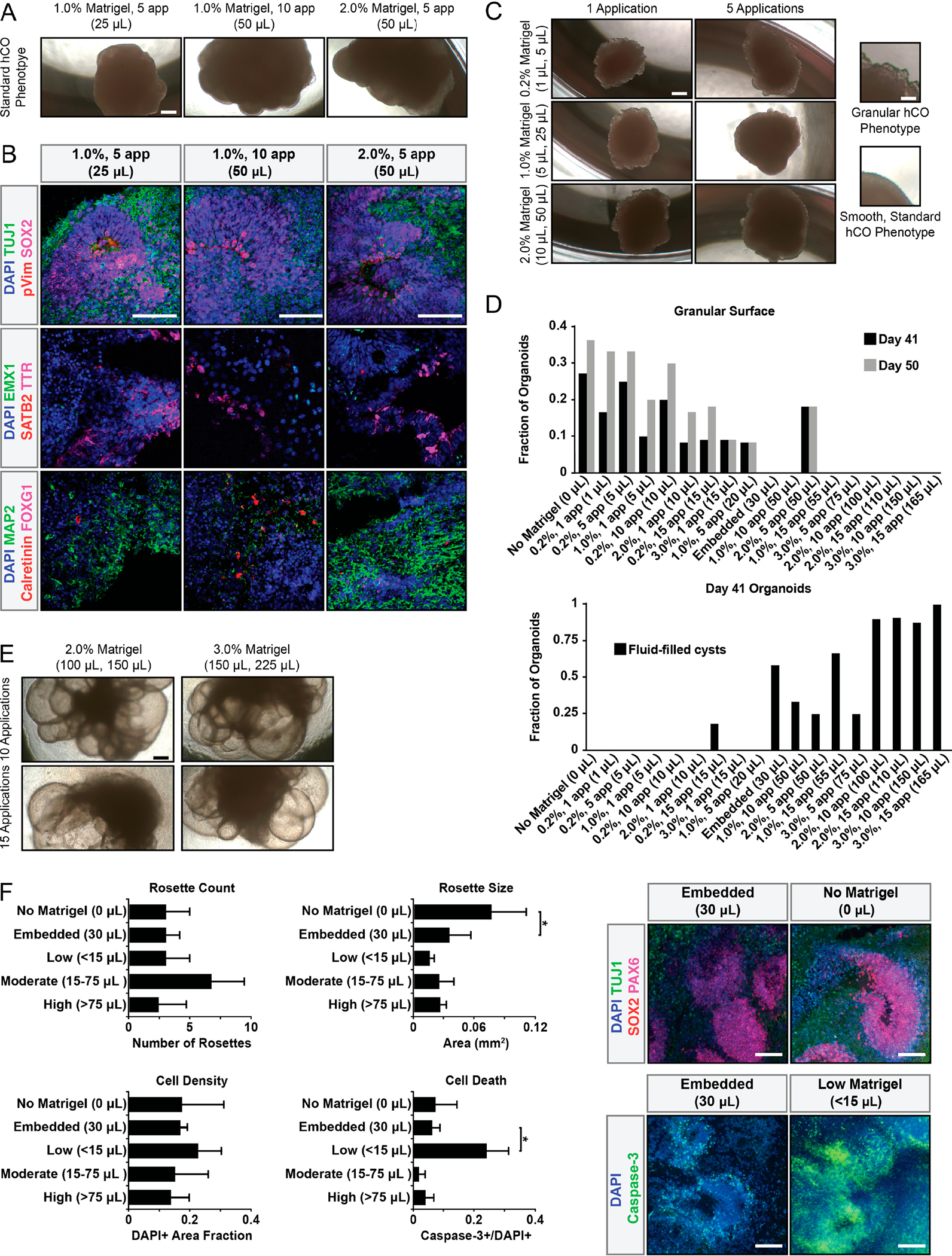
Soluble Matrigel dosage drives three distinct morphological phenotypes. (**A**) Brightfield images of standard hCO phenotype achieved with moderate Matrigel exposure (15–75 μL). Total Matrigel exposure shown in parentheses. Scale bar = 500μm. (**B**) Moderate Matrigel exposure (15–75 μL) drives standard hCO phenotype exhibiting broad cell type composition. Scale bars = 100μm. Total Matrigel exposure shown in parentheses. (**C**) Low Matrigel exposure (<15 μL) creates hCOs with granular surfaces. Total Matrigel exposure shown in parentheses. Scale bars: left = 500μm; right (insets) = 50 μm. (**D**) Granular surface phenotype increases after Matrigel removal from media. (**E**) High Matrigel conditions (>75 μL) yield large fluid-filled cysts. Total Matrigel exposure shown in parentheses (top, bottom). Scale bar = 500 μm. (**F**) Developmental characteristics including rosette development, cell density, and cell death across different conditions. Total Matrigel exposure shown in parentheses. Organoids were generated in a single experiment in separate wells. * *p* < 0.05, via one-way ANOVA with Tukey–Kramer post hoc analysis, n ≥ 2 organoids per condition. Error bars are 95% confidence intervals. Scale bars = 100 μm.

From a simplistic perspective, the presence of growth factors in Matrigel might simply explain the monotonic relationship between size and Matrigel amount. However, in addition to hCO size, the amount of Matrigel affected gross hCO architecture. Three unique morphological hCO phenotypes correlated with low (<15 μL total), medium (15–50 μL total), and high (≥75 μL total) amounts of Matrigel (Figure 2). Medium amounts of Matrigel yielded standard hCO phenotypes, characterized by a smooth, rounded surface and solid tissue [15,22] (Figure 2A). These hCOs contained diverse cell types which are characteristic of whole brain hCOs (Figure 2B). hCOs exposed to low amounts of Matrigel exhibited a thin, granular surface characteristic of non-neural epithelia [19] (Figure 2C). Interestingly, about 10% of hCOs that initially had a smooth surface developed a granular surface after removal of Matrigel relatively late in development (after 6 weeks; day 41) (Figure 2D). hCOs exposed to high levels of Matrigel developed large, fluid-filled cysts with a thin enveloping membrane (Figure 2E). Unlike the fluid-filled cysts of expanded neuroepithelium that sometimes develop in the standard protocol after initial exposure to Matrigel and hCO differentiation media between days 11 and 20 [15,22], these large fluid-filled cysts did not develop into thick, light-impermeable tissue. In addition to morphological analysis, additional hCO characteristics were assessed (Figure 2F). Interestingly, Matrigel absence resulted in larger rosettes which are characteristic of early organoid development [15,22], while cell death increased in only low-Matrigel conditions (<15 μL).

**Figure 3.**
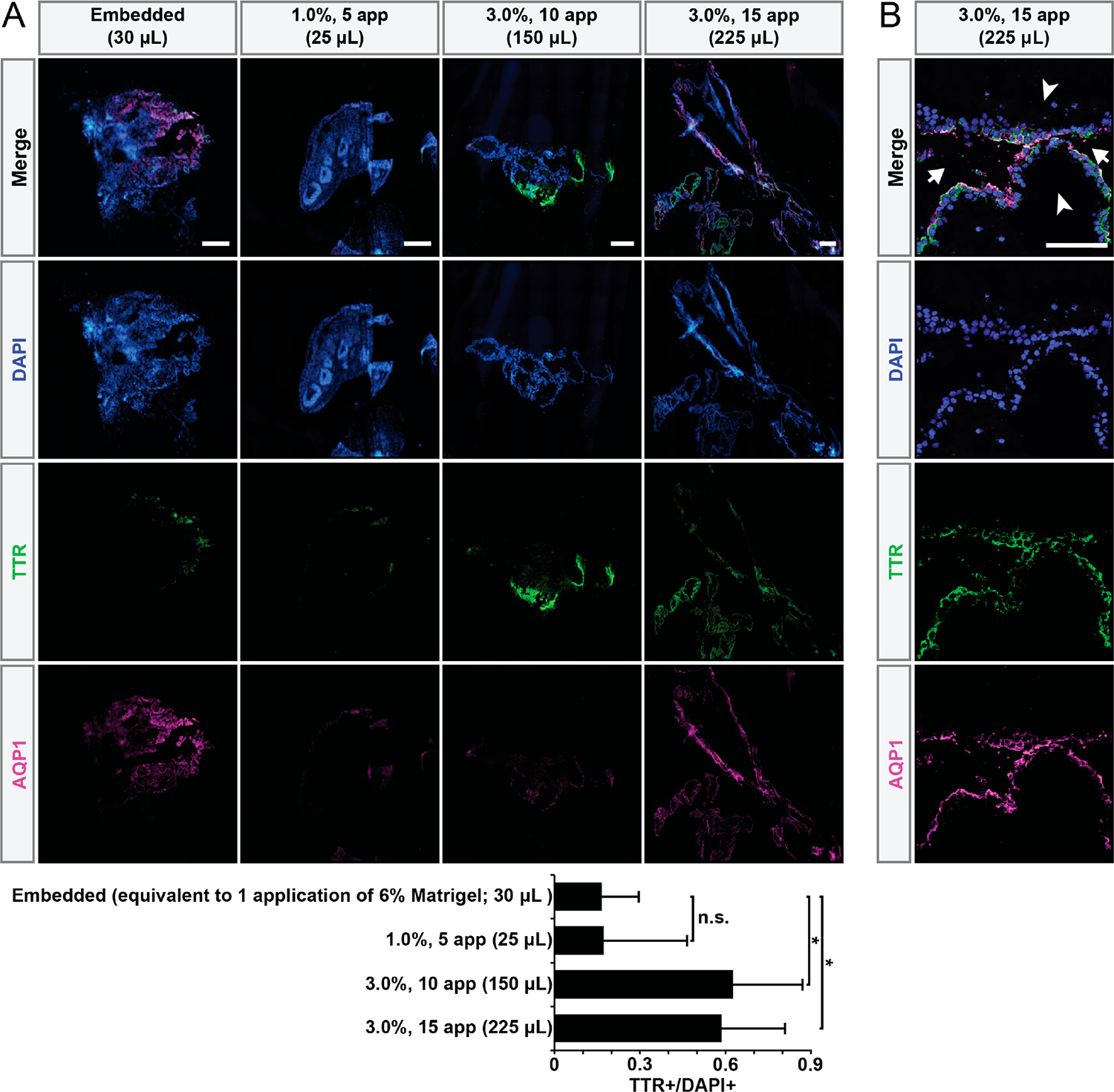
High-concentration Matrigel conditions promote ChP fate. (**A**) Immunostaining confirms hCOs with large fluid-filled cysts exhibit increased number of regions containing ChP cells. Total Matrigel exposure shown in parentheses. Organoids were generated in a single experiment in separate wells. * *p* < 0.05, n.s.: not significant, via one-way ANOVA with Tukey–Kramer post hoc analysis, n ≥ 4 organoids per condition. Error bars are 95% confidence intervals. Scale bars = 500 μm. (**B**) Immunostaining reveals ChP cysts exhibit the expected polarization (apical side: arrows; basal side: arrow heads). Total Matrigel exposure shown in parentheses. Scale bar = 100 μm.

We hypothesized the cystic phenotype would reflect distinct cellular composition. Due to similarities in the morphology of the large fluid-filled hCOs to the choroid plexus (ChP) hCOs generated by Pellegrini and colleagues [2], we hypothesized that high Matrigel exposure increased ChP differentiation. To test this, we immunostained for transthyretin (TTR), a transporter protein produced in the ChP (Figures 3A and S1). Compared to the embedded control, hCOs exposed to high-Matrigel conditions exhibited significantly increased numbers of TTR+ regions. Additionally, we examined whether the fluid-filled cysts of the ChP hCOs exhibited expected apical polarization [2]. We confirmed the correct apical–basal polarity of the ChP hCOs fluid-filled cysts through observing the co-localization of TTR with apical water transporter aquaporin-1 (AQP1) (Figures 3B and S1). Fluid-filled cysts exhibited co-localization of TTR with apical water transporter aquaporin-1 (AQP1), confirming correct apical–basal polarity (Figure 3B).

**Figure 4.**
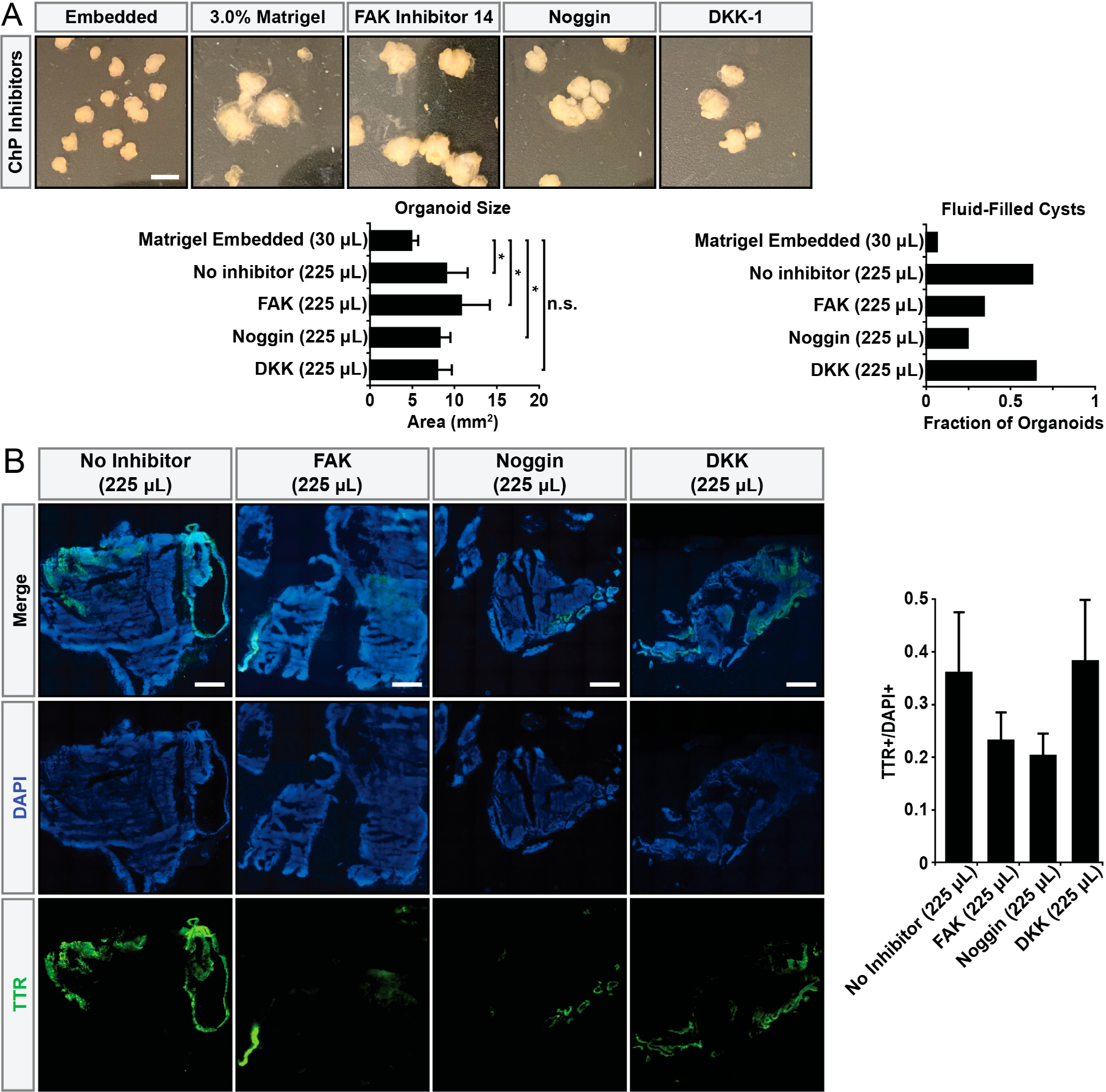
Matrigel promotes ChP differentiation through the BMP4 pathway. (**A**) Brightfield images showing gross morphological differences between hCOs generated in the presence of different pathway inhibitors. Total Matrigel exposure shown in parentheses. Organoids were generated in a single experiment in separate wells. * *p* < 0.05, n.s.: not significant, via one-way ANOVA with Tukey–Kramer post hoc analysis, n ≥ 18 organoids per condition. Error bars are 95% confidence intervals. Scale bar = 500 μm. (**B**) Immunostaining confirms reduction in the number of ChP cells in the presence of Noggin. Total Matrigel exposure shown in parentheses. Error bars are 95% confidence intervals, n ≥ 3 organoids per condition. Scale bars = 500 μm.

To develop a better understanding of how Matrigel might be driving ChP formation, we asked if the pharmacological inhibition of different signal transduction pathways might abrogate this process. Pellegrini and colleagues [2] induced ChP hCOs through pulsing with BMP4, a dorsal telencephalic morphogen, and CHIR, a WNT pathway activator. Interestingly, TGF-β is one of the growth factors present in Matrigel and shares overlapping signaling transduction pathways with BMP signaling [29], so we added an inhibitor of BMP4 signaling, Noggin. Additionally, we asked if the mechanical effects of Matrigel embedding might influence ChP formation. To this end, we used FAK inhibitor 14 [30] to negatively perturb mechanotransduction. Finally, we also used DKK1 to inhibit the WNT pathway. hCOs were generated and cultured in hCO differentiation media with 3% Matrigel in solution, and the inhibitors were added during the initial Matrigel exposure on day 11. While inhibitors did not strongly alter hCO size, the presence of Noggin significantly ablated the appearance of fluid-filled cysts to the level observed in control, Matrigel-embedded hCOs (Figure 4A). We further confirmed this effect through immunostaining for the ChP markers TTR and AQP1 (Figure 4B).

## 4. Discussion

We have shown that hCO growth and development is strongly influenced by Matrigel dosage and delivery method. A traditional hCO phenotype results from moderate levels of soluble Matrigel exposure, while high soluble Matrigel exposure promotes organoid ChP differentiation through the BMP4 pathway, consistent with literature [31,32]. This highlights the tunability of cerebral organoid cell type composition with Matrigel. Through carefully regulating the amount of Matrigel organoids are exposed to or the timing of the exposure, the composition of cerebral organoids can be tuned.

This work reinforces the need for caution when developing and executing protocols, including those beyond cerebral organoids. For example, many non-neural organoid protocols currently require Matrigel, such as those of the liver, heart, and kidney. Similar examinations in those systems may be warranted, even as the specific factors within Matrigel that affect development in different tissues will likely be different. In addition, there are many new competing matrices that have been developed, including Geltrex, LunaGel, ExtragelMatrix, and Cultrex, that will likely have varying compositions in addition to variabilities introduced between lots of these matrices. This work emphasizes the need to understand the dose-dependent effects of any matrix material used in organoid protocols.

Even when Matrigel is not used, this work illustrates the strong change in effect that can occur over relatively small changes in the concentration of a media component. This may explain the variability in organoid phenotypes and quality often observed and reported in the field. Substantial work has been pursued to attempt to replace reagents such as Matrigel [33–46], but it is a complex task to be able to replace all the complex components and functions of such matrices. Perhaps more broadly, the entire media composition for stem cell-derived systems including organoids could be revisited. This is because the media components driving these protocols remain largely derivative of prior base media historically used in cell culture, for example, DMEM media bases. It is unclear if these are still the most appropriate base media compositions for stem cell-derived systems, and if the same effects seen here with Matrigel would be altered or shifted when the base media are changed.

It is important to consider that while these studies shed light on the ability of Matrigel to tune H9-derived hCOs based on dosage, further work should be carried out to explore whether similar effects are observed in other cell lines. Variability between stem cell lines and their propensities to differentiate into different cell and tissue types is well known [47–50]. If upheld in other cell lines, using soluble Matrigel addition for whole brain hCO generation would significantly reduce the time and labor of organoid experiments through circumventing the most time-intensive step in the hCO generation process and enabling higher throughput and more comprehensive experiments to be carried out. It would also be interesting to explore if similar effects are observed when Matrigel is delivered in a solid form, with potential mechanobiological mechanisms also contributing in those cases to organoid development [51].

We also observed two more detailed but interesting phenotypes. In the absence of Matrigel, we observed larger rosettes, which are usually observed early in organoid development and yield larger numbers of rosettes further in development [15,22]. This may suggest that the absence of Matrigel stunts or pauses organoid development. In addition, we observed an increase in cell death only in low-Matrigel conditions (<15 μL) but not in the absence of Matrigel. This may suggest low Matrigel is sufficient to induce additional organoid maturation or induce cell proliferation and differentiation but not sufficient to trophically fully support that development.

Finally, a significant impact of this work is providing a simple method for studying the ChP. Despite its essential functions in producing cerebrospinal fluid and acting as the blood–CSF barrier, the ChP has been understudied due to its relatively inaccessible location within the brain, the surrounding vasculature, and its structural fragility [52–54]. This work provides another valuable tool to address biological questions pertaining to the ChP. Overall, this analysis emphasizes the importance of careful attention to detail required when using, altering, or developing cerebral organoid protocols.

## Supplementary Material

Figure S1

Supplemental Data

## Data Availability

Raw data are available in the Supplemental Materials.

## References

[1] Velasco S; Kedaigle AJ; Simmons SK; Nash A; Rocha M; Quadrato G; Paulsen B; Nguyen L; Adiconis X; Regev A; Individual brain organoids reproducibly form cell diversity of the human cerebral cortex. Nature 2019, 570, 523–527. 31168097 10.1038/s41586-019-1289-x PMC6906116

[2] Pellegrini L; Bonfio C; Chadwick J; Begum F; Skehel M; Lancaster MA Human CNS barrier-forming organoids with cerebrospinal fluid production. Science 2020, 369, eaaz5626. 32527923 10.1126/science.aaz5626 PMC7116154

[3] Mariani J; Simonini MV; Palejev D; Tomasini L; Coppola G; Szekely AM; Horvath TL; Vaccarino FM Modeling human cortical development in vitro using induced pluripotent stem cells. Proc. Natl. Acad. Sci. USA 2012, 109, 12770–12775. 22761314 10.1073/pnas.1202944109 PMC3411972

[4] Qian X; Nguyen HN; Song MM; Hadiono C; Ogden SC; Hammack C; Yao B; Hamersky GR; Jacob F; Zhong C; Brain-Region-Specific Organoids Using Mini-bioreactors for Modeling ZIKV Exposure. Cell 2016, 165, 1238–1254. 27118425 10.1016/j.cell.2016.04.032 PMC4900885

[5] Valiulahi P; Vidyawan V; Puspita L; Oh Y; Juwono VB; Sittipo P; Friedlander G; Yahalomi D; Sohn J-W; Lee YK; Generation of caudal-type serotonin neurons and hindbrain-fate organoids from hPSCs. Stem Cell Rep. 2021, 16, 1938–1952. 10.1016/j.stemcr.2021.06.006 PMC8365029 34242615

[6] Eura N; Matsui TK; Luginbühl J; Matsubayashi M; Nanaura H; Shiota T; Kinugawa K; Iguchi N; Kiriyama T; Zheng C; Brainstem Organoids From Human Pluripotent Stem Cells. Front. Neurosci. 2020, 14, 538. 32670003 10.3389/fnins.2020.00538 PMC7332712

[7] Cederquist GY; Asciolla JJ; Tchieu J; Walsh RM; Cornacchia D; Resh MD; Studer L Specification of positional identity in forebrain organoids. Nat. Biotechnol. 2019, 37, 436–444. 30936566 10.1038/s41587-019-0085-3 PMC6447454

[8] Delepine C; Pham VA; Tsang HWS; Sur M GSK3ß inhibitor CHIR 99021 modulates cerebral organoid development through dose-dependent regulation of apoptosis, proliferation, differentiation and migration. PLoS ONE 2021, 16, e0251173. 33951093 10.1371/journal.pone.0251173 PMC8099055

[9] Amin ND; Kelley KW; Hao J; Miura Y; Narazaki G; Li T; McQueen P; Kulkarni S; Pavlov S; Pasça SP Generating human neural diversity with a multiplexed morphogen screen in organoids. bioRxiv 2023, 1–28. 10.1016/j.stem.2024.10.016 PMC12302009 39642864

[10] Sen D; Voulgaropoulos A; Keung AJ Effects of early geometric confinement on the transcriptomic profile of human cerebral organoids. BMC Biotechnol. 2021, 21, 59. 34641840 10.1186/s12896-021-00718-2 PMC8507123

[11] Lancaster MA; Corsini NS; Wolfinger S; Gustafson EH; Phillips AW; Burkard TR; Otani T; Livesey FJ; Knoblich JA Guided self-organization and cortical plate formation in human brain organoids. Nat. Biotechnol. 2017, 35, 659–666. 28562594 10.1038/nbt.3906 PMC5824977

[12] Kleinman HK; Martin GR Matrigel: Basement membrane matrix with biological activity. Semin. Cancer Biol. 2005, 15, 378–386. 15975825 10.1016/j.semcancer.2005.05.004

[13] Kohen NT; Little LE; Healy KE Characterization of Matrigel interfaces during defined human embryonic stem cell culture. Biointerphases 2009, 4, 69–79. 20408727 10.1116/1.3274061

[14] Hughes CS; Postovit LM; Lajoie GA Matrigel: A complex protein mixture required for optimal growth of cell culture. Proteomics 2010, 10, 1886–1890. 20162561 10.1002/pmic.200900758

[15] Lancaster MA; Renner M; Martin C-A; Wenzel D; Bicknell LS; Hurles ME; Homfray T; Penninger JM; Jackson AP; Knoblich JA Cerebral organoids model human brain development and microcephaly. Nature 2013, 501, 373–379. 23995685 10.1038/nature12517 PMC3817409

[16] Yiangou L; Ross AD; Goh KJ; Vallier L Human Pluripotent Stem Cell-Derived Endoderm for Modeling Development and Clinical Applications. Cell Stem Cell 2018, 22, 485–499. 29625066 10.1016/j.stem.2018.03.016

[17] Fang Y; Eglen RM Three-Dimensional Cell Cultures in Drug Discovery and Development. SLAS Discov. Adv. Sci. Drug Discov. 2017, 22, 456–472. 10.1177/1087057117696795 PMC5448717 28520521

[18] Aisenbrey EA; Murphy WL Synthetic alternatives to Matrigel. Nat. Rev. Mater. 2020, 5, 539–551. 32953138 10.1038/s41578-020-0199-8 PMC7500703

[19] Giandomenico SL; Sutcliffe M; Lancaster MA Generation and long-term culture of advanced cerebral organoids for studying later stages of neural development. Nat. Protoc. 2021, 16, 579–602. 33328611 10.1038/s41596-020-00433-w PMC7611064

[20] Bagley JA; Reumann D; Bian S; Lévi-Strauss J; Knoblich JA Fused cerebral organoids model interactions between brain regions. Nat. Methods 2017, 14, 743–751. 28504681 10.1038/nmeth.4304 PMC5540177

[21] Martins-Costa C; Pham V; Sidhaye J; Novatchkova M; Peer A; Möseneder P; Corsini PS; Knoblich PA Morphogenesis and development of human telencephalic organoids in the absence and presence of exogenous ECM. bioRxiv 2022, 1–28. 10.15252/embj.2022113213 PMC10646563 37842725

[22] Lancaster MA; Knoblich JA Generation of cerebral organoids from human pluripotent stem cells. Nat. Protoc. 2014, 9, 2329–2340. 25188634 10.1038/nprot.2014.158 PMC4160653

[23] Su Y; Besner GE Heparin-binding EGF-like growth factor (HB-EGF) promotes cell migration and adhesion via focal adhesion kinase. J. Surg. Res. 2014, 189, 222–231. 24703506 10.1016/j.jss.2014.02.055 PMC4028394

[24] Hyväri L; Ojansivu M; Juntunen M; Kartasalo K; Miettinen S; Vanhatupa S Focal adhesion kinase and ROCK signaling are switch-like regulators of human adipose stem cell differentiation towards osteogenic and adipogenic lineages. Stem Cells Int. 2018, 2018, 2190657. 30275837 10.1155/2018/2190657 PMC6157106

[25] Rudibaugh TP; Tam RW; Estridge RC; Keung AJ Single cell assessment of human stem cell derived mesolimbic models and their responses to substances of abuse. bioRxiv 2023.

[26] Wei L; Chen C; Ding L; Mo M; Zou J; Lu Z; Li H; Wu H; Dai Y; Xu P; Wnt1 Promotes EAAT2 Expression and Mediates the Protective Effects of Astrocytes on Dopaminergic Cells in Parkinson’s Disease. Neural Plast. 2019, 2019, 1247276. 31582965 10.1155/2019/1247276 PMC6754970

[27] Manfrin A; Tabata Y; Paquet ER; Vuaridel AR; Rivest FR; Naef F; Lutolf MP Engineered signaling centers for the spatially controlled patterning of human pluripotent stem cells. Nat. Methods 2019, 16, 640–648. 31249412 10.1038/s41592-019-0455-2

[28] Yang X; Niu N; Liang C; Wu M-M; Tang L-L; Wang Q-S; Lou J; Song B-L; Zheng W-W; Ma H-P; Stimulation of Epithelial Sodium Channels in Endothelial Cells by Bone Morphogenetic Protein-4 Contributes to Salt-Sensitive Hypertension in Rats. Oxidative Med. Cell. Longev. 2020, 2020, 3921897. 10.1155/2020/3921897 PMC7641672 33194000

[29] La Rosa I; Camargo LS; Pereira MM; Fernandez-Martin R; Paz DA; Salamone DF Effects of bone morphogenic protein 4 (BMP4) and its inhibitor, Noggin, on in vitromaturation and culture of bovine preimplantation embryos. Reprod. Biol. Endocrinol. 2011, 9, 18. 21281523 10.1186/1477-7827-9-18 PMC3042919

[30] Zhou J; Aponte-Santamaría C; Sturm S; Bullerjahn JT; Bronowska A; Gräter F Mechanism of Focal Adhesion Kinase Mechanosensing. PLoS Comput. Biol. 2015, 11, e1004593. 26544178 10.1371/journal.pcbi.1004593 PMC4636223

[31] Hébert JM; Mishina Y; McConnell SK BMP Signaling is required locally to pattern the dorsal telencephalic midline. Neuron 2002, 35, 1029–1041. 12354394 10.1016/s0896-6273(02)00900-5

[32] Watanabe M; Kang Y-J; Davies LM; Meghpara S; Lau K; Chung C-Y; Kathiriya J; Hadjantonakis A-K; Monuki ES BMP4 sufficiency to induce choroid plexus epithelial fate from embryonic stem cell-derived neuroepithelial progenitors. J. Neurosci. 2012, 32, 15934–15945. 23136431 10.1523/JNEUROSCI.3227-12.2012 PMC3505486

[33] Kim S; Min S; Choi YS; Jo S-H; Jung JH; Han K; Kim J; An S; Ji YW; Kim Y-G; Tissue extracellular matrix hydrogels as alternatives to Matrigel for culturing gastrointestinal organoids. Nat. Commun. 2022, 13, 1692. 35354790 10.1038/s41467-022-29279-4 PMC8967832

[34] Giobbe GG; Crowley C; Luni C; Campinoti S; Khedr M; Kretzschmar K; De Santis MM; Zambaiti E; Michielin F; Meran L; Extracellular matrix hydrogel derived from decellularized tissues enables endodermal organoid culture. Nat. Commun. 2019, 10, 5658. 31827102 10.1038/s41467-019-13605-4 PMC6906306

[35] Orlando G; Booth C; Wang Z; Totonelli G; Ross CL; Moran E; Salvatori M; Maghsoudlou P; Turmaine M; Delario G; Discarded human kidneys as a source of ECM scaffold for kidney regeneration technologies. Biomaterials 2013, 34, 5915–5925. 23680364 10.1016/j.biomaterials.2013.04.033

[36] Zachos NC; Kovbasnjuk O; Foulke-Abel J; In J; Blutt SE; de Jonge HR; Estes MK; Donowitz M Human enteroids/colonoids and intestinal organoids functionally recapitulate normal intestinal physiology and pathophysiology. J. Biol. Chem. 2016, 291, 3759–3766. 26677228 10.1074/jbc.R114.635995 PMC4759158

[37] Gilpin SE; Guyette JP; Gonzalez G; Ren X; Asara JM; Mathisen DJ; Vacanti JP; Ott HC Perfusion decellularization of human and porcine lungs: Bringing the matrix to clinical scale. J. Heart Lung Transplant. 2014, 33, 298–308. 24365767 10.1016/j.healun.2013.10.030

[38] Capeling MM; Czerwinski M; Huang S; Tsai Y-H; Wu A; Nagy MS; Juliar B; Sundaram N; Song Y; Han WM; Nonadhesive Alginate Hydrogels Support Growth of Pluripotent Stem Cell-Derived Intestinal Organoids. Stem Cell Rep. 2019, 12, 381–394. 10.1016/j.stemcr.2018.12.001 PMC6373433 30612954

[39] Baptista PM; Siddiqui MM; Lozier G; Rodriguez SR; Atala A; Soker S The use of whole organ decellularization for the generation of a vascularized liver organoid. Hepatology 2011, 53, 604–617. 21274881 10.1002/hep.24067

[40] Saheli M; Sepantafar M; Pournasr B; Farzaneh Z; Vosough M; Piryaei A; Baharvand H Three-dimensional liver-derived extracellular matrix hydrogel promotes liver organoids function. J. Cell. Biochem. 2018, 119, 4320–4333. 29247536 10.1002/jcb.26622

[41] DiMarco RL; Dewi RE; Bernal G; Kuo C; Heilshorn SC Protein-engineered scaffolds for in vitro 3D culture of primary adult intestinal organoids. Biomater. Sci. 2015, 3, 1376–1385. 26371971 10.1039/c5bm00108k PMC9063856

[42] Wylie RG; Ahsan S; Aizawa Y; Maxwell KL; Morshead CM; Shoichet MS Spatially controlled simultaneous patterning of multiple growth factors in three-dimensional hydrogels. Nat. Mater. 2011, 10, 799–806. 21874004 10.1038/nmat3101

[43] Candiello J; Grandhi TSP; Goh SK; Vaidya V; Lemmon-Kishi M; Eliato KR; Ros R; Kumta PN; Rege K; Banerjee I 3D heterogeneous islet organoid generation from human embryonic stem cells using a novel engineered hydrogel platform. Biomaterials 2018, 177, 27–39. 29883914 10.1016/j.biomaterials.2018.05.031

[44] Ng SS; Saeb-Parsy K; Blackford SJ; Segal JM; Serra MP; Horcas-Lopez M; No DY; Mastoridis S; Jassem W; Frank CW; Human iPS derived progenitors bioengineered into liver organoids using an inverted colloidal crystal poly (ethylene glycol) scaffold. Biomaterials 2018, 182, 299–311. 30149262 10.1016/j.biomaterials.2018.07.043 PMC6131727

[45] Cruz-Acuña R; Quirós M; Farkas EA; Dedhia PH; Huang S; Siuda D; García-Hernández V; Miller AJ; Spence JR; Nusrat A; Synthetic hydrogels for human intestinal organoid generation and colonic wound repair. Nat. Cell Biol. 2017, 19, 1326–1335. 29058719 10.1038/ncb3632 PMC5664213

[46] Cruz-Acuña R; Quirós M; Huang S; Siuda D; Spence JR; Nusrat A; García AJ PEG-4MAL hydrogels for human organoid generation, culture, and in vivo delivery. Nat. Protoc. 2018, 13, 2102–2119. 30190557 10.1038/s41596-018-0036-3 PMC7240347

[47] Sun C; Zhang J; Zheng D; Wang J; Yang H; Zhang X Transcriptome variations among human embryonic stem cell lines are associated with their differentiation propensity. PLoS ONE 2018, 13, e0192625. 29444173 10.1371/journal.pone.0192625 PMC5812638

[48] Ortmann D; Brown S; Czechanski A; Aydin S; Muraro D; Huang Y; Tomaz RA; Osnato A; Canu G; Wesley BT; Naive Pluripotent Stem Cells Exhibit Phenotypic Variability that Is Driven by Genetic Variation. Cell Stem Cell 2020, 27, 470–481.e6. 32795399 10.1016/j.stem.2020.07.019 PMC7487768

[49] Volpato V; Webber C Addressing variability in iPSC-derived models of human disease: Guidelines to promote reproducibility. DMM Dis. Model. Mech. 2020, 13, dmm042317. 31953356 10.1242/dmm.042317 PMC6994963

[50] Yang X; Chen D; Sun Q; Wang Y; Xia Y; Yang J; Lin C; Dang X; Cen Z; Liang D; A live-cell image-based machine learning strategy for reducing variability in PSC differentiation systems. Cell Discov. 2023, 9, 53. 37280224 10.1038/s41421-023-00543-1 PMC10244346

[51] Cassel de Camps C; Aslani S; Stylianesis N; Nami H; Mohamed N-V; Durcan TM; Moraes C Hydrogel Mechanics Influence the Growth and Development of Embedded Brain Organoids. ACS Appl. Bio Mater. 2021, 5, 214–224. 10.1021/acsabm.1c01047 35014820

[52] Redzic ZB; Segal MB The structure of the choroid plexus and the physiology of the choroid plexus epithelium. Adv. Drug Deliv. Rev. 2004, 56, 1695–1716. 15381330 10.1016/j.addr.2004.07.005

[53] Lehtinen MK; Bjornsson CS; Dymecki SM; Gilbertson RJ; Holtzman DM; Monuki ES The choroid plexus and cerebrospinal fluid: Emerging roles in development, disease, and therapy. J. Neurosci. 2013, 33, 17553–17559. 24198345 10.1523/JNEUROSCI.3258-13.2013 PMC3818536

[54] Lun MP; Monuki ES; Lehtinen MK Development and functions of the choroid plexus–cerebrospinal fluid system. Nat. Rev. Neurosci. 2015, 16, 445–457. 26174708 10.1038/nrn3921 PMC4629451

