# Supplementary figures and images for: Matrigel Tunes H9 Stem Cell-Derived Human Cerebral Organoid Development
Source: Organoids. Author manuscript; Available in PMC 2024 Jan 9. (PMC10776236; doi:10.3390/organoids2040013)

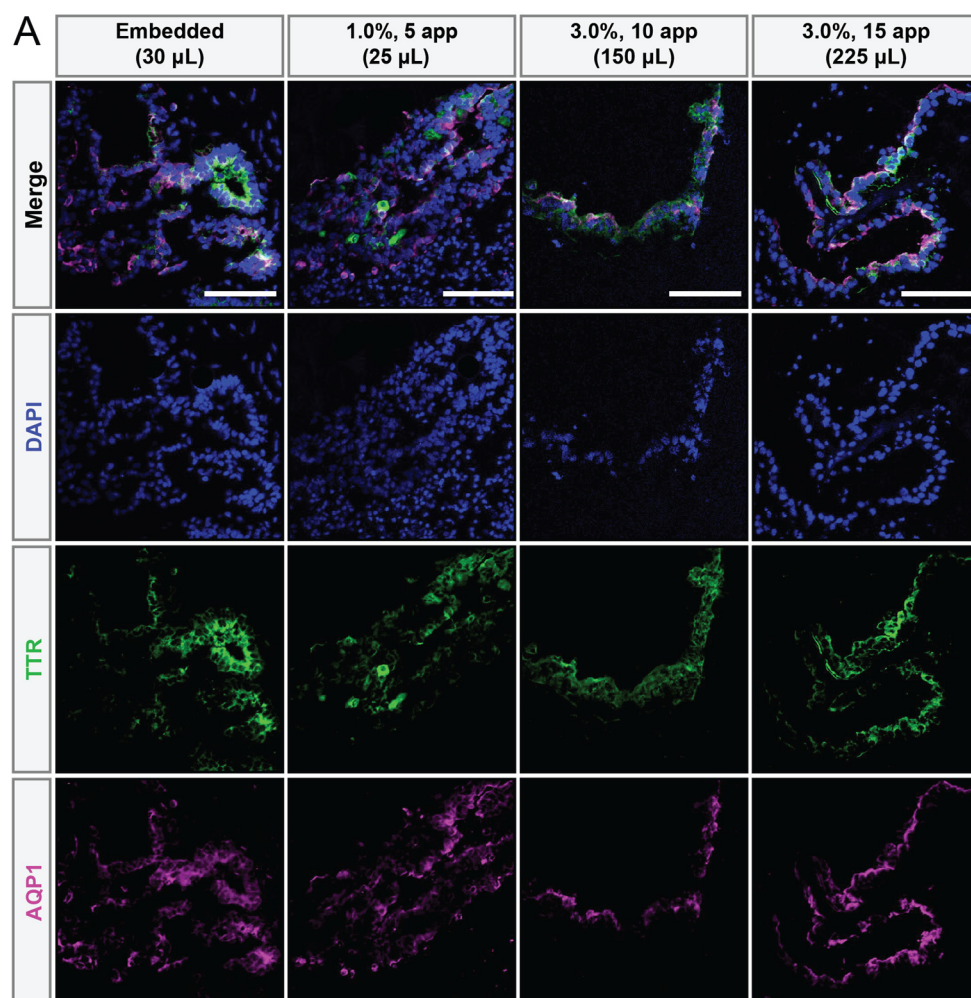

**Figure S1.** High Resolution Images Highlighting TTR+ Region Morphology. Scale bars = 100  $\mu$ m.

Supplement: Figure S1 [file NIHMS1952940-supplement-Figure_S1.pdf]
